# Preconception and contraceptive care for women living with HIV/AIDS attending antiretroviral treatment clinics in Lagos State, Nigeria

**DOI:** 10.4314/ahs.v24i1.5

**Published:** 2024-03

**Authors:** Samuel Oyibo, Atariata Oghenewoke, Mary Odeyemi Balogun, Ugbe Maurice-Joel Ugbe

**Affiliations:** 1 Department of Community Medicine, Faculty of Public Health, University of Ibadan, Nigeria; 2 Department of Public Health, University of Calabar, Calabar, Nigeria

**Keywords:** Preconception care, contraceptive care, HIV/AIDS, women

## Abstract

**Background:**

Women living with HIV/AIDS possess fertility desires similar to their uninfected counterparts, and with advances in health therapies, these women can realistically have and raise uninfected children. Preconception care (PC) is a specialized form of intervention aimed at the prevention, identification, treatment, and management of biomedical, behavioural, and social conditions that militate against safe motherhood and the delivery of healthy offspring.

**Objective:**

The study aimed to assess preconception and contraceptive care among women living with HIV and attending Antiretroviral Therapy Clinics in Alimosho, Lagos State, Nigeria.

**Methods:**

This was a descriptive facility-based cross-sectional study of 383 women of reproductive age living with HIV/AIDS and attending ART clinics in the study area. Probability sampling methods were used in the selection procedures. Data were analyzed using descriptive statistics, Chi-square test, and univariate logistic regression at a 5% level of significance. Stratified and simple random sampling were used in the selection process.

**Results:**

Only 37.4% of respondents received optimal PC services. Being 20-29 years old [OR =1.716 (95% CI: 1.664, 1.769), *p* = 0.020], being 30-39 years [OR =1.514 (95% CI: 0.598, 3.831), *p* = 0.005], tertiary education [OR =8.43. (95% CI: 1.41, 18.5), *p* = 0.020], and being single [OR =2.00 (95% CI: 1.928-2.072), *p* = 0.002] were significantly related to the utilization of contraceptives.

**Conclusion:**

There is a need to provide structure and guidelines for optimal streamlined PC and contraceptive services for women living with HIV/AIDS.

## Introduction

Preconception care is the provision of biomedical, behavioural, and social health interventions to women and couples before conception occurs (Hoyt *et al.*, 2012). It is any preventive, promotive, or curative health program provided to women of childbearing age before pregnancy (at least two years) or between consecutive pregnancies, to improve health-related outcomes for women, newborns, or children under 5 years of age (Dean *et al.*, 2013). PC aims at improving the health status of women, with an ultimate aim of improving maternal and child health, in both the short- and long-term Women of the reproductive age living with HIV are also entitled to preconception care. The goals of preconception care for women living with HIV are to prevent unintended pregnancy, optimize maternal health before pregnancy, improve maternal and fetal outcomes in pregnancy, prevent perinatal HIV transmission, and prevent HIV transmission to an HIV-uninfected sexual partner when trying to conceive(Hoyt *et al.*, 2012). A study indicates that there is an increasing trend in women living with HIV who are becoming pregnant and that many of these conceptions are unintended, thus portraying the underutilization of contraceptives while reflecting an unmet need for PC services (Hoyt *et al.*, 2012).

The Bill & Melinda Gates Foundation (2021) reported that the global Maternal Mortality Ratio (MMR) was at 152 deaths per 100,000 live births, a little above the 2019 report of 151 deaths per 100,000 live births. This data is a pointer that by 2030, there will be 133 maternal deaths per 100,000 live births, almost double the Sustainable Development Goals (SDGs) target, if proper measures are not taken to tackle it (Bill & Melinda Gates Foundation, 2021). Itis estimated that about half of global maternal deaths occur in Sub-Saharan Africa, mostly due to complications such as eclampsia, bleeding, infections, and obstructed labour (Cofie *et al*, 2015). However, the Bill & Melinda Gates Foundation (2021) reported that there were 302 maternal deaths per 100,000 live births as of 2020 in sub-Saharan Africa. The majority of these deaths would be largely prevented if the patronage of skilled birth attendants was high. For instance, Mustapha *et al.* (2020) found that Nigeria has the highest maternal mortality rate in the region. This was affirmed by records showing that as of 2020, there were 324 maternal deaths per 100,000 live births in Nigeria (Bill & Melinda Gates Foundation, 2021). There is also a growing concern that approximately 810 women die every day due to pregnancy and childbirth-related complications- mainly from preventable or treatable causes like infectious diseases and pre-and postpartum complications (World Health Organization, 2021). As the world is currently off-track to achieving SDG 3.1, urgent actions need to be taken to improve maternal and child survivals through comprehensive HIV/AIDS care for mothers.

Approximately 214 million women of reproductive age in developing countries have unmet contraceptive needs (World Health Organization, 2020). One in five pregnancies in Nigeria are unplanned and one in ten Nigerian women have aborted an unwanted pregnancy; while among women living with HIV in sub-Saharan Africa, 35%-65% of pregnancies are unplanned and 62% of preceding pregnancies are also unplanned (Kikuchi *et al*, 2011). This increases the risk of vertical transmission of HIV from mother to child. In essence, nearly 500,000 children globally are infected with HIV through mother-to-child transmission annually and in Nigeria, 90% of HIV infections among children are transmitted from mother to child (United Nations Program on HIV/AIDS, 2021). Most studies have not considered these two concepts, which has left a knowledge gap in the area of understanding how the paucity of both preconception and contraceptive care among Women Living with HIV/AIDS affect maternal and child health outcomes. It is on this premise that this present study was undertaken to identify and improve PC and contraceptive care services among women of childbearing age living with HIV/AIDS in Lagos, Nigeria.

## Methods

### Study area

The study sites for the research were antiretroviral treatment clinics situated in Alimosho L.G.A, Lagos State, Southwest, Nigeria. Antiretroviral treatment clinics are clinics where women living with HIV access treatment, care and support, they are located across various health facilities, both government and private. In Alimosho, there are 53 antiretroviral treatment centres in total, 39 Primary Health centres, 1 General hospital and 13 private centres (APIN 2018).

### Study design

A facility-based descriptive cross-sectional study design was used to assess preconception and contraceptive care services provided to women of reproductive age living with HIV and receiving antiretroviral care within the facilities in Alimosho Local Government Area.

### Study population

The study population were women of reproductive age (15 to 49 years) living with HIV/AIDS and receiving antiretroviral care at treatment clinics in Alimosho Local Government Area.

### Sample size determination

The sample size was determined using Fischer's formula as cited in Charan and Biswas (2013) for estimating the sample size of Cross-sectional studies- $n=\frac{z^{2}p\;(1-p)}{d^{2}}$ n= minimum sample-size; d = degree of accuracy set at 0.05 level of precision (5%); *z* = standard normal deviation set at 1.96 normal interval at 5% (95% confident interval); p = 47.7% (Practice of preconception care among women in antenatal clinic at Enugu state university teaching hospital) by Nwogu-Ikojo et al. (2010); $1-p=1-0.477=0.523-n=\frac{1.96^{2}\times 0.477\times 0.523}{0.05^{2}}n=383$

Making provision for 10% nonresponse, the minimum sample size was:426

### Sampling procedure

The sampling technique was multi-staged

**Stage 1:** Facilities were first stratified into Government and Private treatment centres. Thereafter they were divided based on their status into primary and secondary health facilities. Then one facility from each cadre was selected using a simple random sampling technique.

**Stage 2:** Proportionate sampling was used to determine the number of respondents per facility; this was done to have an equal representation of respondents per facility, and to avoid selection bias.

(The same for B, C, and D.) where, $A=\frac{a}{a+b+c+d}\times 421$ respondent per facility.

a = population of women living with HIV within the reproductive age receiving treatment in government primary health facility.

b = population of women living with HIV within the reproductive age receiving treatment in government secondary health facility.

c = population of women living with HIV within the reproductive age receiving treatment in a private primary health facility.

d = population of women living with HIV within the reproductive age receiving treatment in a private secondary health facility.


$$A=\frac{550}{550+815+220+180}\;x\;426=133$$

$$B=\frac{815}{550+815+220+180}\;x\;426=197$$

$$C=\frac{220}{550+815+220+180}\;x\;426=53$$

$$D=\frac{180}{550+815+220+180}\;x\;426=43$$

Lastly, simple random sampling with replacement was done to select participants for the study during each clinic visit. For every patient who came into the facilities on their clinic days, we assigned them unique identifiers. These numbers were folded and placed in a container, shaken and the numbers were picked. Questionnaires were administered to those whose numbers were picked.

### Data collection procedures

Data were collected by the primary investigator and with the aid of three trained research assistants; the research assistants were trained on the purpose of the study, the skill for the administration of the questionnaire and the need to maintain privacy and confidentiality throughout data collection. The research assistants were supervised by the primary investigator.

### Instruments for data collection

An interviewer-administered questionnaire was used in collecting information from respondents. Questions from previously validated instruments were adapted and modified. The instruments include the checklist for preconception care by the American College of Obstetricians and Gynaecologists (Family Planning National Training Center, 2019); and a preconception/ prenatal family health History Questionnaire by the March of Dimes Foundation (Yumpu, 2013). The semi-structured questionnaire had four sections; Section A elicited information on Socio-demographic characteristics and Obstetric History; section B elicited information on preconception care services received; section C elicited information on contraceptive care services received, and Section D elicited information on contraceptive utilization. The questionnaire was initially in English but later translated to Yoruba and back-translated to English to ensure that its original meaning was retained. Administration of the questionnaire was done for 2 months on Wednesdays and Fridays during Antenatal and drug pick-up days.

### Method of data analysis

Data were collected, manually sorted out and entered into the Statistical Package for the Social Sciences (SPSS)version 20, numbers were used to code each response and the open-ended questions were grouped and recorded. Preconception care services had a list of 12 components with a “Yes” or “No” answer, respondents that answered yes to the core preconception care service known as, nutritional health care, screening for chronic conditions, screening for HIV/STIs, family planning counselling, and assessment for potential pregnancy were said to have received optimal preconception care. Contraceptive care services received had a standard list of various contraceptive methods from the Nigeria Demographic Health Survey (NDHS), with a “yes” or ‘No” option attached to them, to know various contraceptive services received. The contraceptive utilization section had four questions including questions containing standard contraceptive methods extracted from NDHS 2018, it was divided into contraceptive method currently used and contraceptive method ever used. The categorical variables were described using descriptive statistics such as percentages, means and standard deviations, and relationships between two categorical variables were determined through the Chi-Square test. Univariate logistic regression was used to identify factors associated with contraceptive utilization. The tests were carried out at a 5% level of significance.

### Ethical consideration

Ethical approval to conduct the study was obtained from Lagos State University Teaching Hospital (LREC/06/10/1600). Written informed consent was obtained from respondents and they were informed that participation is voluntary and no consequences for non-participation. Names and addresses were not obtained from the respondents to ensure confidentiality. Ethical protocols that concern social science research such as confidentiality of data, beneficence to participants, and voluntariness were duly observed by the researchers in line with the 1964 Declaration of Helsinki and its later amendments.

## Results

### Socio-demographic characteristics of respondents

Data were retrieved from 383 respondents giving a 90% response rate. Table 1 shows the demographic characteristics of the study's respondents. The mean age was 30 ±1.3 years, more than one-third (46.2%) of the respondents were between the age of 30-39 years. Regarding the level of education, 51.4% had no formal education, 46.2% were traders by occupation, 30.3% were single, 67.4% were from a monogamous family, and 68.3% of respondents that were married had a monogamous family type. Table 1 also shows the result for Obstetric and family history. Slightly over half of the respondents (50.7%) had ever been pregnant, 55.2% of respondents that had ever been pregnant did not plan their last pregnancy and 54.1% did not receive antenatal care. One-third of the respondents that received antenatal care had their ANC at primary health care facilities, 44.8% of the respondents who had ever been pregnant delivered their baby at Traditional birth attendants (TBA) homes.

**Table 1:** Demographic and obstetric characteristics of respondents (n=3

Variables	Frequency	Percent
Age		
15-19 years	71	18.5
20-29 years	100	26.1
30-39 years	177	46.2
40-49 years	35	9.2
Mean age (±SD)	30 (±1.3)	
Level of education		
Primary	78	20.4
Secondary	55	14.4
Tertiary	53	13.8
No formal education	197	51.4
Occupation		
Trader	177	46.2
Teacher	14	3.7
Artisan	140	36.5
Civil servants	52	13.6
Marital status		
Not married	282	73.6
Ever married	101	26.4
Ever been pregnant		
Yes	194	50.7
No	189	49.3
Last pregnancy planned (n=194)		
Yes	87	44.8
No	107	55.2
Received ANC (n=194)		
Yes	89	45.9
No	105	54.1
The facility where ANC was received (n=89)		
Primary health care	30	33.7
Secondary health care	21	23.6
Tertiary health care	10	11.2
TBA	28	31.5
Place of delivery (n=194)		
Hospital	52	26.8
Home	37	19.1
TBA	87	44.8
Mission home	18	9.2
The outcome of last pregnancy (n=194)		
Delivered	183	94.0
Miscarriage	11	6.0

*SD= Standard deviation

Table 2 shows preconception care services received by respondents, 367 (95.8%) respondents were screened for HIV/STI which was the most received preconception care service, 182 (47.5%) received family planning counselling, and 180 (47%) received nutritional healthcare. Advice on environmental health issues 12 (3.1%) was the least received preconception care service. In essence, optimal preconception care was however not received by the majority of respondents.

**Table 2:** Preconception care services received(n=383)

Preconception care services received	Yes N (%)	No N (%)
Screening for HIV/STI	367 (95.8)	16 (4.2)
Family planning counseling	182 (47.5)	201 (52.5)
Nutritional health care	180 (47.0)	203 (53.0)
Advice on psychoactive substances	176 (45.9)	207 (54.1)
Vaccination against rubella virus	146 (38.1)	237 (61.9)
Screening for a chronic condition	133 (34.7)	250 (65.3)
Mental health care	127 (33.2)	256 (66.8)
Advice on IPV	110 (28.7)	273 (71.3)
Assessment for potential pregnancy	102 (26.6)	281 (73.4)
Genetic counseling	96(25.0)	287 (75.0)
Screening for FGM	86 (22.5)	297 (75.5)
Advice on environmental health issues	12 (3.1)	371 (96.9)

*Multiple responses allowed

Table 3 shows the percentage of respondents that received contraceptive methods. Male condoms were the most commonly used (43.0%) while female sterilization was the least utilized contraceptive method (1.1%).

**Table 3:** Contraceptive utilization among women living with HIV (n = 383)

Contraceptive Utilization	Frequency	Percent
Ever had sexual intercourse		
Yes	318	83.0
No	65	17.0
Contraceptive method ever used. (n=262)		
Male condoms	105	40.0
Pills	48	18.3
Injectable	41	16.0
Implants	31	12.0
Lactational amenorrhea (LAM)	21	8.0
Intrauterine device (IUD)	13	4.7
Female sterilization	3	1.1
The contraceptive method currently used. (n=262)		
Male condoms	112	43.0
Pills	55	21.0
Injectables	49	19.0
Implants	16	6.1
Lactational amenorrhea (LAM)	17	6.4
Intrauterine device (IUD)	10	3.8
Female sterilization	3	1.1

Table 4 indicates the distribution of chi-square p-values to establish the association between the sociodemographic characteristics of the study's respondents and optimal preconception care services received. The sociodemographic factors significantly associated with preconception care services received in the study population include age, χ^2^ (3, N= 383) = 3.957, *p*=0.047; and marital status, χ^2^ (1, N= 383) = 4.451, *p*=0.035. Table 4 also presents the distribution of chi-square p-values to establish the association between sociodemographic characteristics and utilization of contraceptive services by the study's respondents. Age, χ^2^ (3, N= 383) = 12.361, *p*=0.002; marital status, χ^2^ (1, N= 383) = 6.994, *p*=0.008; and level of education, χ^2^ (3, N= 383) = 5.594, *p*=0.009 were statistically significantly associated with utilization of contraceptive services by women living with HIV/AIDS in the study area.

**Table 4:** Chi-Square analysis of Sociodemographic characteristics, receipt of optimal Preconception care services and contraceptives utilization

Variables	Preconception Care Services Categories			Contraceptive Care utilization Categories		
	Yes	No	*p*-Value	Yes	No	*p*-Value
	N (%)	N (%)		N (%)	N (%)	
Age						
15-19 years	25 (35.2)	46 (64.8)	0.047*	17 (23.9)	54 (76.1)	0.002*
20-29 years	41(41.0)	59 (59.0)		60 (60)	40(40)	
30-39 years	99 (55.9)	78 (44.1)		116 (65.5)	61 (34.5)	
40-49 years	10 (28.6)	25 (71.4)		12 (34.3)	23 (65.7)	
Level of Education						
Primary	50 (64.1)	28 (35.9)	0.446	17 (21.8)	61 (78.2)	0.009*
Secondary	18 (32.7)	37 (67.3)		20 (36.4)	35 (63.6)	
Tertiary	20 (37.7)	33 (62.3)		9 (16.9)	44 (83.1)	
No Formal Education	109 (54.7)	88 (44.3)		55 (20.5)	142(79.5)	
Occupation						
Trader	115 (64.9)	62 (35.1)	0.092	56(31.6)	121(68.4)	0.176
Teacher	10 (71.4)	4 (28.6)		5 (35.7)	9 (64.3)	
Artisan	36 (25.7)	104(74.3)		61(43.5)	79 (56.5)	
Civil Servants	19 (36.5)	33 (63.5)		33 (63.4)	19 (36.6)	
Marital Status						
Not Married	189 (67.0)	93 (33.0)	0.035*	179(63.5)	103(36.5)	0.008*
Ever Married	45 (44.5)	56 (55.5)		39(38.6)	62 (61.4)	

* =Statistical significance based on *p*-Value < 0.05

Table 5 shows the logistic regression analysis of the demographic correlates associated with optimal preconception care services received, and utilization of contraceptives. Respondents aged 20-29 years [OR =1.716 (95% CI: 1.664, 1.769), *p* = 0.020] were 1.7 times more likely than other age groups to utilize contraceptives. Also, respondents aged 30-39 years [OR =1.514 (95% CI:1.044, 3.831), *p* = 0.005] were 1.514 times more likely than respondents in the oldest age group to utilize contraceptives. In terms of education, respondents who had attained tertiary education [OR =8.43. (95% CI: 1.41, 18.5), *p* = 0.020] were significantly more likey than those with no formal education to utilize contraceptives. In terms of marital status, respondents who were single [OR =2.00. (95% CI: 1.928-2.072), *p* = 0.002] were twice more likely to utilize contraceptives than their married counterparts.

**Table 5:** Logistic Regression showing the relationship between socio-demographic characteristics, optimal preconception care services received, and utilization of contraceptives

Variables	Preconception Care Services Categories			Contraceptive Care utilization Categories		
	OR	95% CI	*p*-Value	OR	95% CI	*p*-Value
Age						
15-19 years	1.514	0.598-3.831	0.462	1.468	1.409-1.526	0.066
20-29 years	0.996	0.122-8.129	0.997	1.716	1.664-1.769	0.020*
30-39 years	1.545	0.257-9.274	0.06	1.514	1.044-3.831	0.005*
40-49 years	*1	-	-	*1	-	-
Level of Education						
Primary	--	--	--	1.97	0.46, 8.33	0.360
Secondary	--	--	--	3.30	0.83, 13.11	0.091
Tertiary	--	--	--	8.43	1.41, 18.5	0.020*
No Formal Education	--	--	--	*1	-	-
Marital Status						
Not Married	1.204	0.140-10.332	0.866	2.00	1.928-2.072	0.002*
Ever Married	*1	-	-	-	-	-

OR= Odds Ratio; CI= Confidence Interval; *p*-value= Probability value; *=Statistical significance based on *p*-Value < 0.05; *1= Reference group

## Discussion

The study aimed at assessing preconception and contraceptive utilization among women living with HIV/AIDS in Antiretroviral Treatment Clinic at Alimosho Local Government Area Lagos State.

### Preconception care services among respondents

Results from the study showed that overall, less than half of respondents received optimal preconception care services. Similarly, an Ethiopian study found that only 6.4% of women received the full package of PC services in the community (Habte *et al*, 2021). Another study by Amaje *et al.* (2022) found a low utilization of PC services by women living with HIV in Orioma, Ethiopia. Poor overall utilization of PC services may be attributed to the people's level of awareness, cultural norms, or fear of stigma and discrimination owing to the sensitive nature of their health conditions. The study also revealed that over 90% of respondents who received PC services were more likely to be screened for HIV/STI. Similarly, Ekem *et al.* (2018) found HIV care services to be the most utilized service among respondents in the South West, Nigeria. Also, Demisse *et al.* (2019) found that among many PC services, HIV services were majorly utilized (92.7%) by respondents in North Shewa, Ethiopia. In contrast, Habte *et al.* (2021) found that folic acid supplementation was the commonest PC service received by women in their Ethiopian study. Varying views may be attributed to the diverse percentages of pregnant women in each study's target population. In this present study, few (26.6%) of the respondents we reassessed for potential pregnancy which also shows a dearth in service provision of core preconception care service across ART clinics in the study area.

Genetic counselling which was one of the least received preconception care services is an important component of preconception care as reported in a study on awareness of genetic counselling in Lagos by Yama *et al.* (2019). In a classic work, Hecht (1987) elicits that improving the knowledge of HIV/AIDS prevention and management among women of reproductive age is by implication important for genetic counsellors who may encounter infected pregnant women in the course of their clinical practice. There is a dearth of information linking the importance of genetic counsellors to HIV care services. Thus there is a need for emphasis on assessing genetic counsellors for women of childbearing age living with HIV to scale up the prevention of mother-to-child transmission. In this present study, younger respondents (30-39 years) were about 1.56 times more likely than respondents in other age groups to receive optimal preconception services.

In a retrospective study, similar findings were made by Loutfy *et al.* (2014) who reported that those aged over 40 years were less likely than the youths to utilize PC services. Other studies have found a significant relationship between younger and middle ages with increased use of PC services and have argued that it is logical that such a relationship exists (Nakayiwa *et al*, 2006; Nattabi *et al.*, 2009; Oosterhoff *et al.*, 2008). However, although women between 20 and 35 are usually counselled, women in their advanced ages may still desire to conceive and may proceed with conceiving without counselling and proper PC care or may not conceive at all due to a lack of proper education.

### Contraceptive care services received among respondents

One of the strategies adopted by the joint United Nations Program on HIV/AIDs to eliminate mother-to-child transmission globally by 2023 is the uptake of contraceptive use among women of reproductive age living with HIV. However, some of these contraceptive services are not available across ART clinics. The study revealed that contraceptive services received across selected facilities cuts across, including female sterilization, Intrauterine device, implants, injectables, and provision of male condoms. The provision of condoms was the most received contraceptive service as this was readily available across ART clinics. Female sterilization was the least contraceptive service received with only 1.1% of respondents receiving it. This is similar to the findings by Kahansim *et al.* (2020) about the rise and fall of female sterilization in Jos, Nigeria. It was observed that there was a decline in the acceptance of female sterilization, from a peak of 36.1% in 1992 to 1.4% in 2018. This may be attributed to the fact that sterilization is permanent and many women may not want to take the risk of becoming infertile since the desire to procreate may resurface.

Furthermore, our results show contraceptive utilization at 30%. This is similar to the study carried out by Basha et al. (2021) among women living with HIV in the Amhara region of Ethiopia where contraceptive utilization was at 30.3%. Results of this study also showed that about 43% of respondents were currently using only condoms as a contraceptive method; this is similar to the result from the study conducted by Adeleye *et al.* (2019) on reproductive plans and utilization of contraceptives among women living with HIV, with over 50% of respondents currently using condoms only as a contraceptive method. This is because bar abstinence, the use of condoms is the next safest method of preventing HIV and other STIs. This present study found that respondents aged 30-39 years were 1.5 times more likely than respondents in the oldest age group to utilize contraceptives. This is consistent with findings from an Eritrean study by Idris et al. (2021) which reported that younger women living with HIV/AIDS were 1.6 times more likely to utilize contraceptives. This difference between age groups may be due to the expectations of women regarding the physiological cessation of menstruation and the fear of side effects with increasing age. It has also been suggested that older women are more likely to be separated or divorced from their partners and might not be sexually active to utilize contraceptives (Karraker *et al.*, 2011; Spangler *et al.*, 2014).

This study also found that respondents who had attained tertiary education were over 8 times more likely than those with no formal education to utilize contraceptives. This is consistent with studies by Feleke *et al.* (2013) and Nyarko (2015) which found that women living with HIV with higher educational attainment were more likely to utilize contraceptives. Although a study like that of Idris et al. (2021) had contrasting views, it is only logical that people with higher educational attainment are better enlightened hence our findings. Lastly, our study found that single women were twice more likely to utilize contraceptives than their married counterparts. This is in contrast to the studies by Idris *et al.* (2021) and Adeyemi *et al.* (2016) which found that married women were twice more likely to utilize contraceptives than singles. Our findings may be due to statistical reasons such that there are significantly higher numbers of single respondents than married respondents in the study. On the other hand, our findings may be because single women are more cautious about multiple sexual partners than their married counterparts.

### Strengths and limitations

This study adopted and used validated instruments, which ensured that the results were reliable. The study also has some limitations. ART clinics assessed in course of the study were only government and private primary and secondary facilities. ART clinics in tertiary health facilities were not assessed as there was no tertiary health facility in the LGA where the study was carried out. Secondly, this was a cross-sectional study hence results must be read with caution.

## Conclusion

The study found that adequate optimal preconception and contraceptive care services were not been received by women living with HIV across ART clinics in Alimosho L.G. A, although there are needs for them. Based on the findings, there is a need for integration of family planning services with ART services provided to women living with HIV/AIDS. Also, emphasis should be placed by health care service providers on core preconception care services to have optimal service delivery of preconception care among women living with HIV. There should be the adoption of preconception care as a core strategy in the fight to eliminate mother-to-child transmission of HIV.
